# 晚期*EGFR*阳性NSCLC患者PD-L1表达特点及其与EGFR-TKIs治疗疗效关系的真实世界研究

**DOI:** 10.3779/j.issn.1009-3419.2023.101.09

**Published:** 2023-03-20

**Authors:** Ran CHEN, Xiang GAO, Fudong XU, Shucai ZHANG, Li MA, Bo HU

**Affiliations:** ^1^441000 襄阳，湖北医药学院附属襄阳市第一人民医院肿瘤科（陈然，胡波）; ^1^Department of Oncology, Xiangyang No. 1 People's Hospital, Hubei University of Medicine, Xiangyang 441000, China; ^2^101149 北京，首都医科大学附属北京胸科医院，北京市结核病胸部肿瘤研究所肿瘤内科（陈然，高翔，张树才，马丽）; ^2^Department of Medical Oncology, Beijing Chest Hospital, Capital Medical University, Beijing Tuberculosis and Thoracic Tumor Research Institute, Beijing 101149, China; ^3^101149 北京，首都医科大学附属北京胸科医院，北京市结核病胸部肿瘤研究所病理科（徐福东）; ^3^Department of Pathology, Beijing Chest Hospital, Capital Medical University, Beijing Tuberculosis and Thoracic Tumor Research Institute, Beijing 101149, China

**Keywords:** 肺肿瘤, EGFR突变, PD-L1表达, 靶向治疗, Lung neoplasms, Epidermal growth factor receptor mutation, Programmed cell death ligand 1 expression, Targeted treatment

## Abstract

**背景与目的** 晚期表皮生长因子受体（epidermal growth factor receptor, EGFR）阳性非小细胞肺癌 (non-small cell lung cancer, NSCLC）患者的一线治疗首选EGFR酪氨酸激酶抑制剂（epidermal growth factor receptor tyrosine kinase inhibitors, EGFR-TKIs）。晚期驱动基因阴性，程序性死亡配体1（programmed cell death ligand 1, PD-L1）阳性或高表达的NSCLC患者一线治疗推荐免疫检查点抑制剂（immune checkpoint inhibitors, ICIs）单药治疗或ICIs联合化疗。所以不同PD-L1表达水平的EGFR阳性晚期NSCLC患者的一线治疗策略值得进一步探究。既往众多研究提示PD-L1的表达明显受EGFR突变情况的影响，PD-L1表达很有可能与EGFR-TKIs耐药机制相关。本研究旨在分析晚期EGFR阳性NSCLC患者PD-L1表达特点及其与EGFR-TKIs疗效的关系。**方法** 以159例EGFR阳性初治晚期NSCLC患者（其中包含141例EGFR敏感突变患者）为研究对象，分析159例患者相关临床病理特征与PD-L1表达的关系及141例EGFR敏感突变患者EGFR-TKIs治疗疗效的的相关影响因素。**结果** 所纳入159例患者PD-L1表达按肿瘤细胞阳性比例分数（tumor proportion score, TPS）分类：阴性（TPS<1%）占47.2%，低表达（1%≤TPS<50%）占32.1%，高表达（TPS≥50%）占20.7%。癌细胞病理形态学分类实体为主型患者更容易出现PD-L1高表达（高表达占比52.9%，组间差异P<0.0001）。PD-L1阴性表达、低表达、高表达患者一线接受一代EGFR-TKIs治疗的中位无进展生存期分别为12.4个月、10.5个月和3.7个月，三者间差异有明显统计学意义（P<0.0001）；EGFR-TKIs治疗期间，有肺部病灶放疗史相对于无肺部病灶放疗史有更长的无进展生存期获益（中位无进展生存期：17.0个月 vs 9.3个月，P<0.0001）。**结论** 晚期EGFR阳性NSCLC患者PD-L1的表达水平与癌细胞病理形态学分类显著相关。PD-L1高表达NSCLC患者接受EGFR-TKIs疗效明显较差。靶向治疗期间联合肺部病灶放疗可以显著延长无进展生存期。

## Abstract (en)

**Background and objective** Epidermal growth factor receptor-tyrosine kinase inhibitors (EGFR-TKIs) is the first choice for first-line treatment of advanced patients with EGFR positive non-small cell lung cancer (NSCLC). For advanced NSCLC patients with negative drive gene and positive programmed cell death ligand 1 (PD-L1) or highly expressed NSCLC patients, the first-line treatment recommends immune checkpoint inhibitors (ICIs) monotherapy or ICIs combined chemotherapy. Therefore, the first-line treatment strategy for advanced NSCLC patients with EGFR positive at different PD-L1 expression levels is worth further exploring. Many previous studies have suggested that the expression of PD-L1 is obviously affected by EGFR mutation, and the expression of PD-L1 is likely to be related to the mechanism of EGFR-TKIs resistance. The purpose of this study was to analyze the expression characteristics of PD-L1 in patients with advanced EGFR positive NSCLC and its relationship with the efficacy of EGFR-TKIs. **Methods** 159 patients with newly diagnosed advanced NSCLC with EGFR positive (including 141 patients with EGFR sensitive mutations) were enrolled to analyze the relationship between clinicopathological characteristics and PD-L1 expression. The factors affecting the therapeutic effect of EGFR-TKIs in 141 patients with EGFR sensitive mutations were also explored. **Results** The PD-L1 expression of the included patients was classified according to the tumor promotion score (TPS): negative (TPS<1%) accounted for 47.2%, low expression (1%≤TPS<50%) accounted for 32.1%, and high expression (TPS≥50%) accounted for 20.7%. Among them, patients with solid predominant cancer cell pathomorphology classification are more likely to have high expression of PD-L1, accounting for 52.9% (P<0.0001). The median progression-free survival (mPFS) of patients with PD-L1 negative expression, low expression and high expression who received first generation EGFR-TKIs treatment were 12.4 months, 10.5 months and 3.7 months, respectively, with significant statistical difference (P<0.0001). During the EGFR-TKIs treatment, patients with a history of radiotherapy for lung lesions have a longer PFS benefit than those without a history of radiotherapy (mPFS: 17.0 months vs 9.3 months, P<0.0001). **Conclusion** The expression level of PD-L1 in advanced EGFR positive NSCLC patients was significantly correlated with the pathomorphology classification of cancer cells. The efficacy of EGFR-TKIs in patients with PD-L1 overexpression NSCLC was significantly poor. PFS can be significantly prolonged during targeted therapy combined with radiotherapy of lung lesions.

2021年权威期刊CA Cancer J Clin发布了2020年全球范围里36种恶性肿瘤的发病率和死亡率数据，结果显示肺癌目前仍是发病率和死亡率均位居前列的恶性肿瘤，2020年，肺癌新发病例约220万，占确诊恶性肿瘤患者的11.4%，发病率现位居第二，仅次于乳腺癌；死亡人数180万，死亡率18%，仍居首位^[1]^。初诊肺癌患者中有超过2/3为III期-IV期，丧失手术治疗机会，预后较差，5年总生存率不超过5%。表皮生长因子受体（epidermal growth factor receptor, EGFR）突变作为NSCLC最为常见的突变亚型之一，占NSCLC人群的1/3^[2]^，东亚地区、女性、无吸烟史的肺腺癌患者占比更高。EGFR酪氨酸激酶抑制剂（EGFR-tyrosine kinase inhibitors, EGFR-TKIs）给这部分EGFR阳性的晚期NSCLC患者带来了明显的生存获益，目前已经成为一线的标准治疗^[3]^。近年来，随着免疫检查点抑制剂（immune checkpoint inhibitors, ICIs）在肺癌治疗中获得诸多成功，国内外多项临床指南对晚期EGFR/间变性淋巴瘤激酶（anaplastic lymphoma kinase, ALK）野生型NSCLC患者的一线治疗给出了明确推荐：程序性死亡配体1（programmed cell death ligand 1, PD-L1）阳性表达，特别是高表达患者可选择ICIs单药治疗，如阿特珠单抗、帕博利珠单抗等^[4⇓-6]^。那么，真实世界中存在的EGFR突变型且PD-L1阳性表达甚至高表达的患者该如何抉择，目前尚缺乏循证医学证据。不同PD-L1表达状态的EGFR阳性患者对接受EGFR-TKIs的治疗反应如何?有小样本临床研究^[7]^显示：PD-L1高表达的患者接受一代EGFR-TKIs疗效欠佳。EGFR突变对PD-L1的表达水平有何影响?近年来众多基础及临床研究结果^[8,9]^显示：EGFR突变导致PD-L1表达上调还是下调仍是众说纷纭、并无定论。不同EGFR突变亚型的PD-L1表达水平是否有差异?EGFR阳性NSCLC患者的PD-L1表达有何特点?这些是都是与临床息息相关的问题。本研究拟通过对晚期EGFR突变型NSCLC患者的临床病理特征及接受EGFR-TKIs治疗疗效进行真实世界的研究，对以上问题做出初步回答。

## 1 材料与方法

### 1.1 患者情况

本研究通过首都医科大学附属北京胸科医院临床研究伦理审批（No.YJS-2021-031）。以首都医科大学附属北京胸科医院2019年1月1日-2021年12月31日期间收治的EGFR突变型晚期NSCLC患者为研究对象。纳入标准：（1）经病理学或细胞学明确为NSCLC；（2）经突变扩增系统-聚合酶链式反应（amplification refractory mutation system-polymerase chain reaction, ARMS-PCR）技术检测明确为EGFR突变型（突变类型包括：19del、L858R、20INS、L861Q、G719X、S768I）；（3）根据美国癌症联合会（American Joint Committee on Cancer, AJCC）第八版肿瘤原发灶-淋巴结-转移（tumor-node-metastasis, TNM）分期，确定为IIIB期-IV期或局部晚期无法行手术治疗的患者；（4）纳入人群中19del、L858R两种突变类型患者一线治疗为第一代EGFR-TKIss单药（包含吉非替尼、埃克替尼、厄洛替尼）或包含有第一代EGFR-TKIs的联合治疗且疗效纳入评价，其余EGFR突变亚型患者治疗方式为其他，不纳入治疗疗效评价；（5）EGFR-TKIs治疗期间，有肺部病灶放疗史的患者放疗方式为适形调强放疗（intensity-modulated radiation therapy, IMRT），肿瘤靶区射线剂量为50 Gy-60 Gy；（6）美国东部肿瘤协作组（Eastern Cooperative Oncology Group, ECOG）评分0分-2分；（7）根据实体瘤疗效评价标准（Response Evaluation Criteria in Solid Tumours, RECIST）1.1版本评价要求，至少有1个以上的可评估疗效的病灶。排除标准：（1）合并存在ALK融合、c-ros原癌基因1酪氨酸激酶（c-ros oncogene 1 receptor tyrosine kinase, ROS1）重排等其他敏感突变；（2）存在第二原发肿瘤。

### 1.2 检测方法

#### 1.2.1 EGFR突变检测

利用ARMS技术检测肿瘤患者组织学或细胞学标本中EFGR基因突变类型。由首都医科大学附属北京胸科医院病理科提供石蜡切片标本，用QIAamp DNA试剂盒定量提取石蜡切片组织中肿瘤细胞的DNA^[10]^。通过ARMS-PCR技术检测肿瘤组织中EGFR突变类型，厦门艾德公司ADx-ARMS试剂盒检测29种突变类型，设内部和外部质控样本、阳性对照和阴性对照。

#### 1.2.2 PD-L1表达检测

从病理科借取组织或细胞学蜡块，每例标本连续切片3张，防脱载玻片捞片。用热修复方式进行抗原修复。使用22C3抗体（PD-L1 IHC 22C3 pharmDx, Dako）进行免疫组化染色，经显色、苏木精对比染色，分化、脱水、透明、封片。PD-L1判读标准：PD-L1表达依据肿瘤细胞阳性比例分数（tumor proportion score, TPS）判读染色结果，定义为部分或完整膜染色的肿瘤细胞占样品中存在的所有肿瘤细胞的百分比。PD-L1表达按TPS=1%、TPS=50%的cutoff分类为表达阴性、低表达、高表达（图1）。两名高年资病理医师采用双盲判读法，结果不一致者重新阅片；结果相差明显者，共同镜下阅片。

**图1:**
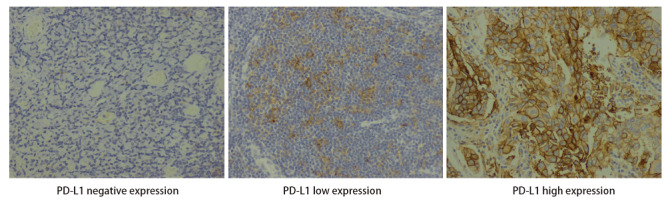
免疫组化染色展示肿瘤PD-L1表达水平（×200）

### 1.3 资料收集

查阅医院电子病历系统，收集患者的人口学、吸烟史、临床病理学特征、基因突变状态、突变亚型、PD-L1表达水平、初诊时血液癌胚抗原（carcino-embryonic antigen, CEA）水平、脑转移状态、靶向期间胸部病灶放疗史、治疗及预后生存资料。

### 1.4 疗效评估及随访

患者开始治疗前和治疗期间定期行影像学检查（颅脑磁共振、胸腹部计算机断层扫描、浅表淋巴结彩超、骨扫描等）。依据RECIST 1.1标准进行疗效评估。末次随访时间为2022年5月31日。无进展生存期（progression-free survival, PFS）定义为从接受EGFR-TKIs治疗开始至观察到疾病进展（progressive disease, PD）或因任何原因导致死亡的时间，随访终点尚未发生进展者定义为删失。

### 1.5 统计学方法

采用SPSS 23.0软件进行统计学分析。PD-L1的表达水平与临床病理学特征间的关系使用χ^2^检验或Fisher精确检验进行率的比较。使用Kaplan-Meier方法进行生存分析，Log-rank检验进行组间比较。P值均为双侧检验，P<0.05为差异有统计学意义。将可能影响EGFR-TKIs治疗后PFS的变量因素整合，使用Cox回归风险比例模型对这些因素进行单因素和多因素回归分析。

## 2 结果

### 2.1 患者临床病理特征

本研究共纳入患者159例，其中一线接受一代EGFR-TKIs治疗的患者（含19del、L858R）共141例；EGFR-TKIs联合治疗方式包括EGFR-TKIs联合化疗（铂类±培美曲塞）、EGFR-TKIs联合抗血管生成药物（重组人血管内皮抑制素注射液或贝伐珠单抗注射液）。患者的年龄、性别、吸烟史、病理类型、PD-L1表达情况、是否合并TP53共突变、治疗方式相关资料详见表1。初诊时EGFR突变亚型为19del患者76例，L858R患者65例，其他突变亚型（包含20INS、L861Q、G719X、S768I）18例；初诊时血液CEA水平正常者59例，CEA水平异常升高者100例；癌细胞病理形态学亚型中腺泡亚型为主型患者75例，乳头或微乳头亚型为主患者33例，实体亚型为主型34例，其余未进行病理形态学检测分类；根据RECIST 1.1进行疗效评价：部分缓解（partial response, PR）、疾病稳定（stable disease, SD）、PD的患者病例数分别为83例、68例、8例（表1）。

**表1:** 所有患者临床病理学特征（n=159）

Characteristic	n (%)
Age, median (range)(yr) <65 ≥65	64 (32-86) 85 (53.5) 74 (46.5)
Gender Male Female	62 (39.0) 97 (61.0)
Smoking history Never Current or former	112 (70.4) 47 (29.6)
Pathologic type Adenocarcinoma Squamous-cell carcinoma Adenosquamous carcinoma	151 (95.0) 4 (2.5) 4 (2.5)
PD-L1 expression (TPS) TPS<1% 1%≤TPS<50% TPS≥50%	75 (47.2) 51 (32.1) 33 (20.7)
Primary EGFR mutation 19del L858R Others	76 (47.8) 65 (40.9) 18 (11.3)
CEA level (>6 ng/mL) Normal Elevated	59 (37.1) 100 (62.9)
TP53 comutation Yes No Unknown	37 (34.6) 70 (65.4) 52 (32.9)
Morphology of cancer cell Acinar predominant Papillary/Micropapillary predominant Solid predominant Unknown	75 (47.2) 33 (20.8) 34 (21.4) 17 (10.6)
Brain metastasis Yes No	31 (19.5) 128 (80.5)
Treatment EGFR-TKIs monotherapy EGFR-TKIs combination therapy	124 (78.0) 35 (22.0)
Radiotherapy Yes No	22 (15.6) 137 (86.2)
RECIST response PD SD PR	8 (5.0) 68 (42.8) 83 (52.2)

PD-L1: programmed cell death ligand 1; TPS: tumor proportion score; CEA: carcinoembryonic antigen; TP53: tumor protein p53; EGFR-TKIs: epidermal growth factor receptor tyrosine kinase inhibitors; RECIST: Response Evaluation Criteria in Solid Tumors; PD: progressive disease; SD: stable disease; PR: partial response; 19del: exon 19 deletion; L858R: 21 exon L858R mutation.

### 2.2 EGFR阳性NSCLC患者PD-L1表达水平与临床病理特征关系

在所有入组人群中，按TPS=1%和TPS=50%将PD-L1表达分类为PD-L1阴性表达（TPS<1%）、低表达（1%≤TPS<50%）和高表达（TPS≥50%）（表2）。PD-L1经免疫组化染色后，不同表达水平的染色效果如图1所示。统计结果显示：PD-L1表达（阴性/低表达/高表达）在性别、年龄、吸烟史、EGFR突变亚型、合并TP53突变状态、血液CEA水平及初诊时脑转移状态中均无显著统计学差异，而在癌细胞病理形态亚型分类中有统计学差异（图2）。在癌细胞病理形态亚型分类中：癌细胞病理形态学亚型实体为主型患者更倾向于出现PD-L1高表达，占比52.9%，P<0.0001（表2）。

**表2:** 所有患者PD-L1表达水平与临床病理学特征关系（n=159）

Variable	n	PD-L1 expression [n (%)]			P
		TPS<1%	1%≤TPS<50%	TPS≥50%	
Age (yr)					0.8310
<65	85	42 (49.4)	26 (30.6)	17 (20.0)	
≥65	74	33 (44.6)	25 (33.8)	16 (21.6)	
Gender					0.9967
Male	62	29 (46.8)	20 (32.3)	13 (21.0)	
Female	97	46 (47.4)	31 (32.0)	20 (20.6)	
Smoking					0.7380
Never	112	55 (49.1)	35 (31.3)	22 (19.6)	
Current or former	47	20 (42.6)	16 (34.0)	11 (23.4)	
EGFR mutation					0.0878
19del	76	32 (42.1)	30 (39.5)	14 (18.4)	
L858R	65	31 (47.7)	16 (24.6)	18 (27.7)	
Others	18	12 (66.7)	5 (27.8)	1 (5.5)	
TP53 comutation					0.7860
Yes	37	14 (37.8)	14 (37.8)	9 (24.3)	
No	70	31 (44.3)	25 (35.7)	14 (20.0)	
Unknown	52	30 (57.7)	12 (23.1)	10 (19.2)	
Morphology of cancer cell					<0.0001
Acinar predominant	75	47 (62.7)	20 (26.7)	8 (10.7)	
Papillary/Micropapillary predominant	33	20 (60.6)	10 (30.3)	3 (9.1)	
Solid predominant	34	5 (14.7)	11 (32.4)	18 (52.9)	
Unknown	17	3 (17.6)	10 (58.8)	4 (23.6)	
CEA level					0.0507
Normal	59	21 (35.6)	21 (35.6)	17 (28.8)	
Elevated	100	54 (54.0)	30 (30.0)	16 (16.0)	
Brain metastasis					0.0587
Yes	31	9 (29.0)	12 (38.7)	10 (32.3)	
No	128	66 (51.6)	39 (30.5)	23 (17.9)	

**图2:**
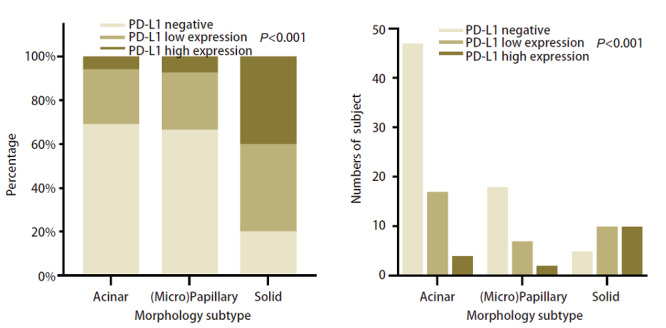
肿瘤细胞不同病理学形态亚型PD-L1表达情况。 A：百分比展示；B：绝对数展示。

### 2.3 EGFR-TKIs疗效的影响因素分析

将纳入生存分析的141例EGFR敏感突变患者的一般资料及临床病理资料，包括：年龄、性别、吸烟史、病理类型、EGFR突变亚型、初诊时血液CEA水平、合并相关基因突变状态、治疗方式、是否合并胸部病灶放疗及肿瘤PD-L1表达进行了单因素和多因素回归分析及Kaplan-Meier生存Log-rank检验。单因素分析结果显示（表3）：患者EGFR突变亚型、EGFR-TKIs给药方式（EGFR-TKIs单药或EGFR-TKIs单药联合其他）、有无靶病灶放疗史、PD-L1表达能显著影响EGFR-TKIs治疗的疗效及PD风险；然后将单因素分析中P<0.02的因素纳入多因素Cox回归分析进行校正。多因素Cox回归分析结果提示PD-L1表达及EGFR-TKIs期间肺部病灶放疗可显著影响EGFR-TKIs治疗的疗效及疾病进展风险（表3）。

**表3:** EGFR敏感突变人群中，影响PFS的单因素和多因素回归分析（n=141）

Variable	Univariate analysis			Multivariate analysis	
	HR (95%CI)	P		HR (95%CI)	P
Gender		0.523			
Male	1.121 (0.790-1.589)				
Female					
Age (yr)		0.848			
≥65	0.967 (0.683-1.368)				
<65	Reference				
Smoking		0.810			
Yes	1.047 (0.721-1.521)				
No	Reference				
TP53 comutation		0.987			
Yes	0.997 (0.648-1.532)				
No	Reference				
CEA level		0.714			
Elevated	0.933 (0.644-1.352)				
Normal	Reference				
Morphology of cancer cell					
Acinar predominant	0.961 (0.563-1.641)	0.884			
Papillary/Micropapillary predominant	1.070 (0.570-2.008)	0.833			
Solid predominant	Reference				
EGFR mutation		0.013			0.055
19del	0.645 (0.455-0.913)			0.708 (0.497-1.008)	
L858R	Reference			Reference	
Brain metastasis		0.219			
Yes	1.307 (0.853-2.001)				
No	Reference				
PD-L1 expression					
TPS≥50%	7.175 (4.331-11.886)	<0.001		7.08 (4.227-11.889)	<0.001
1%≤TPS<50%	1.236 (0.833-1.836)	0.293		1.212 (0.814-1.804)	0.344
TPS<1%	Reference			Reference	
Treatment		0.018			0.742
EGFR-TKIs combination therapy	0.621 (0.418-0.923)			0.916 (0.543-1.544)	
EGFR-TKIs monotherapy	Reference			Reference	
Radiotherapy		0.005			0.016
Yes	0.505 (0.312-0.816)			0.522 (0.306-0.887)	
No	Reference			Reference	

#### 2.3.1 PD-L1表达与EGFR-TKIs疗效呈负相关

PD-L1表达按TPS=1%、50%将患者分类为PD-L1阴性表达、PD-L1低表达和PD-L1高表达，结果显示相对于PD-L1高表达组，PD-L1阴性组和PD-L1低表达组均有着更好的EGFR-TKIs治疗疗效[mPFS: 12.4个月 vs 10.5个月 vs 3.7个月，P<0.0001；客观缓解率（overall response rate, ORR）：90.38% vs 54.35% vs 34.38%，P<0.0001；≥1年EGFR-TKIs治疗应答率：55.56% vs 45.65% vs 3.13%，P<0.0001]（图3）。

**图3:**
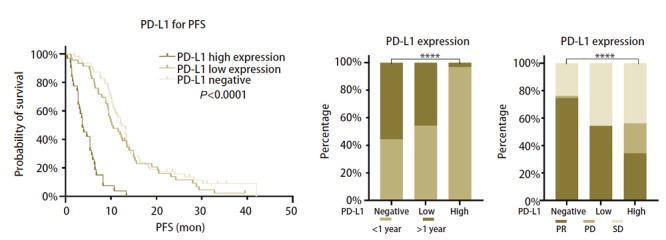
PD-L1表达水平与EGFR-TKIs疗效的关系

#### 2.3.2 EGFR-TKIs治疗期间行肺部病灶放疗可明显提高EGFR-TKIs治疗疗效

EGFR-TKIs治疗期间行肺部病灶放疗患者相对于无放疗史患者有着更好的EGFR-TKIs治疗疗效（mPFS：17个月 vs 9.3个月，HR=0.380，95%CI：0.2623-0.7955，P<0.0001；ORR：86.36% vs 56.30%，P=0.0265；≥1年EGFR-TKIs治疗应答率：90.91% vs 31.09%，P<0.0001）（图4）。

**图4:**
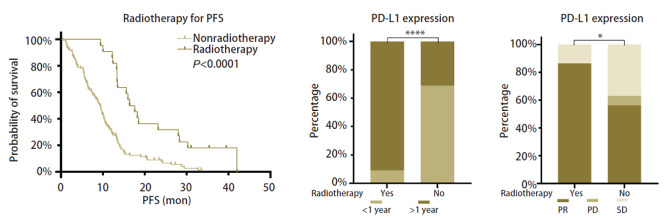
EGFR-TKIs治疗期间肺部病灶放疗史与EGFR-TKIs疗效的关系

## 3 讨论

程序性死亡受体1（programmed cell death 1, PD-1）作为免疫球蛋白超家族1型跨膜糖蛋白，主要表达于活化的T细胞表面。其配体PD-L1，作为B7超家族成员，在T淋巴细胞、B淋巴细胞、树突状细胞、巨噬细胞及多种恶性肿瘤组织中广泛表达^[11]^。PD-L1和肿瘤突变负荷（tumor mutational burden, TMB）、微卫星不稳定/错配修复缺陷（microsatellite instability/mismatch repair, MSI-H/dMMR）目前已在美国食品药品监督管理局（Food and Drug Adminitration, FDA）获批作为泛实体瘤免疫治疗伴随诊断生物标记物应用于筛选ICIs治疗优势人群并可从一定程度上预测疗效^[5,12⇓-14]^，提示PD-L1表达对ICIs治疗有着正向预测作用，现已经广泛应用于肿瘤临床，特别是在驱动基因阴性的NSCLC患者的ICIs治疗疗效预测中有着举足轻重的地位。然而针对于驱动基因阳性的晚期NSCLC患者，2020年中国临床肿瘤学会（Chinese Society of Clinical Oncology, CSCO）和2021版美国国立综合癌症网络（National Comprehensive Cancer Network, NCCN）指南均提示：一线靶向治疗的地位不可动摇，尽管部分患者PD-L1表达水平较高，但ICIs单药治疗效果欠佳^[15]^。PD-L1在驱动基因阴性NSCLC患者ICIs治疗中的预测价值是值得肯定的，在驱动基因阳性NSCLC患者中的预测价值却有待商榷^[16,17]^。

在寻找影响EGFR-TKIs疗效的影响因素及EGFR-TKIs耐药机制的探索中，目前已有大量的研究进行报道，主要包括EGFR依赖性和EGFR非依赖性。其中EGFR依赖性主要包括有EGFR通路中继发EGFR外显子20 T790M突变^[18]^、EGFR C797S/L729突变^[19]^及相关罕见突变，如D761Y^[20]^、L747S^[21]^等。EGFR非依赖性耐药机制主要包括磷脂酰肌醇3-激酶（phosphatidylinositol 3-kinase, PI3K）/蛋白激酶B（protein kinase B, PKB/Akt）/哺乳动物雷帕霉素靶蛋白（mammalian target of rapamycin, mTOR）通路激活^[22]^、大鼠肉瘤/丝裂原活化蛋白激酶（rat sarcoma-mitogen-activated protein kinase, Ras-MAPK）通路激活^[23]^、间质上皮转换因子（mesenchymal epithelial transition factor, MET）扩增^[24]^、人表皮生长因子受体2（human epidermal growth factor receptor 2, HER-2）扩增^[25]^等。目前针对以上部分耐药机制进行的药物研发，例如第四代EGFR-TKIs、MET抑制剂等已经应用于临床，也从一定程度上缓和了EGFR-TKIs耐药后的局面，但整体效果不甚完美。而关于PD-L1表达在EGFR-TKIs耐药机制中的探索目前相关的研究并不多，包括一些小样本的临床回顾性研究分析和个案报道，已经看到了PD-L1表达预测EGFR-TKIs疗效的趋势^[26]^。因此本研究在真实世界中，探索了EGFR阳性NSCLC患者人群中PD-L1的表达特点及其与EGFR-TKIs疗效的关系。

本研究中，159例EGFR阳性NSCLC患者的人口流行病学及临床病理学特征显示：纳入患者中女性（61.0%）、不吸烟（70.4%）、病理类型为腺癌（95.0%），这与既往流行病学研究报道的EGFR突变多发生于亚裔、不抽烟的女性肺腺癌患者的结论一致^[27⇓-29]^。有关于EGFR突变亚型的分布特点方面：EGFR的突变亚型中19del和L858R是最常见且与EGFR-TKIs疗效最为相关的两种突变亚型，在突变人群中占比80%-90%^[30]^，也与本研究统计结果一致（88.7%）。长期以来，CEA作为一种泛肿瘤标记物，广泛应用于肿瘤临床诊疗中，用以预测预后、评价疗效等。我们发现：所纳入EGFR阳性患者中，大部分患者（62.9%）初诊时血液中CEA水平升高，这与既往研究结果吻合。CEA的表达与EGFR突变密切相关，EGFR突变率会随着CEA水平的增高而增高^[31]^。这可能与EGFR突变后下游信号通路激活促进了抑制肿瘤细胞凋亡的发生，进而提高了作为凋亡产物的CEA蛋白水平的表达。TP53基因突变是一种在绝大部分癌症中广泛存在的基因突变类型，在NSCLC中约占50%^[32]^，在EGFR阳性的NSCLC中突变频率偏低（25%-30%）^[33]^，与本研究统计结果接近（34.6%）。并且有临床前研究^[34]^证据表明TP53突变与EGFR-TKIs反应性之间存在联系，特别是吉非替尼等一代EGFR-TKIs。所以我们将是否合并TP53突变也纳入了EGFR-TKIs的疗效分析中。

关于EGFR突变对PD-L1表达的影响在既往的基础研究和临床研究中的结果并不一致。但多数研究结果倾向于认为EGFR突变可提高PD-L1阳性表达率。Akbay等^[35]^于 2013年通过小鼠模型发现EGFR突变可诱导PD-L1表达，促进免疫抑制性微环境的形成，而PD-1单抗能明显抑制EGFR突变肿瘤的生长并延长生存期。Azuma等^[8]^通过检测164例NSCLC手术切除标本PD-L1表达水平，首次报道EGFR突变具有较高的PD-L1免疫组化评分，且PD-L1高表达预示着预后不良。然而，也有研究得出与此不同的结论，Takada等^[36]^通过对441例手术切除的肺腺癌标本进行免疫组化染色，发现PD-L1表达中有21%与野生型EGFR显著相关。一项汇总分析^[37]^纳入18项研究的3,969例患者，发现EGFR突变型NSCLC的PD-L1表达较野生型更低，比值比（odds ratio, OR）为0.59（95%CI: 0.39-0.92, P<0.02）。

在EGFR突变阳性患者PD-L1表达特点的研究中，既往有研究^[38]^显示：在泛NSCLC患者人群中PD-L1阳性表达率为39.9%-53.1%。也有研究^[38⇓-40]^显示：在EGFR突变型肺癌患者中PD-L1阳性表达率为53.6%-71.9%。本研究中EGFR突变阳性NSCLC患者PD-L1阳性表达率为52.8%（84/159）。我们的研究结果与上述既往报道结果相比：（1）比既往已报道的泛NSCLC人群PD-L1阳性表达率稍高，究其原因可能是本研究中研究对象均为EGFR阳性患者，不包含EGFR野生型患者。因EGFR突变可能导致PD-L1阳性表达，故导致本研究人群中PD-L1阳性率稍高。（2）比既往已报道EGFR阳性人群占比稍低，究其原因可能是本研究中患者临床分期较晚，均为IIIB期-IV期，不包含早、中期患者。已有研究^[41,42]^证实：临床分期（包括TNM分期）对PD-L1阳性表达有确切影响。所以，可能是本研究中晚期病例比例偏高，所以影响了PD-L1的阳性表达率。当我们对PD-L1的表达进行EGFR的两种常见敏感突变亚型分层时：我们发现19del亚型和L858R亚型PD-L1表达并无差异（P=0.1708），这与既往的研究结果并不统一。Jain等^[43]^发现EGFR L858R亚型PD-L1表达高于EGFR 19del亚型。Song等^[44]^发现EGFR 19del亚型PD-L1表达高于EGFR L858R亚型，同时也有研究^[45]^发现：这两种突变亚型之间的PD-L1表达并无差异，与本研究结果相似。腺癌细胞的病理亚型包括：实体型、乳头微乳头型、腺泡型及贴壁型，不同亚型的癌细胞恶性程度不同，从而导致了NSCLC患者的预后差异^[46]^。本研究对所纳入人群病理形态学亚型与PD-L1表达之间的关系进行了分析。我们发现：相对于其他亚型，实体为主型患者PD-L1阳性表达较高，高表达率更加突出（P值均<0.0001），与既往研究结果^[43,47]^一致。同时我们也发现：初诊时合并脑转移也会显著影响PD-L1的表达，初诊时合并脑转移的患者更倾向出现PD-L1表达阳性，这与前人的研究^[41,42]^也比较吻合。

在针对一线接受EGFR-TKIs治疗的敏感突变人群中我们对影响EGFR-TKIs疗效的各种因素进行了探索，最终的多因素回归分析结果显示：PD-L1的表达对EGFR-TKIs的疗效影响显著，特别是PD-L1高表达人群，治疗效果显著下降，mPFS仅3.7个月，显著低于阴性表达人群（12.4个月）和低表达人群（10.5个月）。首都医科大学附属北京胸科医院张卉教授团队有一项关于PD-L1高表达与靶向治疗关系的临床研究^[26]^，对2例PD-L1高表达且EGFR阳性的NSCLC患者接受EGFR-TKIs治疗的过程进行了追踪，结果发现这2例患者疗效及预后极差，最终总生存期（overall survival, OS）均未超过半年，此结果与我们的研究结果也完全一致。近年来，EGFR阳性晚期NSCLC患者的一线EGFR-TKIs联合治疗方式的研究也有了诸多开展，其中包括EGFR-TKIs联合抗血管生成药物、联合化疗、联合局部放疗、联合同步放化疗等多种联合方式。其中部分联合方案的临床研究正在进行中，也有部分联合模式的临床研究已经有了良好的结果。最近的SINDAS研究（NCT02893332）^[48]^，纳入了133例初治晚期EGFR敏感突变的非脑转移患者，一线治疗分为EGFR-TKIs单药和EGFR-TKIs联合胸部放疗两组，接受一代EGFR-TKIs（包含埃克替尼、吉非替尼、厄洛替尼）治疗，随访23.6个月，主要观察终点为PFS，次要观察终点为OS和治疗相关性不良反应（treatment related adverse reactions, TRAEs），结果发现：EGFR-TKIs单药组和联合组mPFS分别为12.5个月和20.2个月（P<0.001）；中位OS分别为17.4个月 vs 25.5个月（P<0.001）。此项临床研究与本研究对照分组一致，研究结果也与本研究类似。近年来还有EGFR-TKIs联合立体定向放疗（stereotactic body radiation therapy, SBRT）的临床研究也在开展中，后续结果值得我们期待。所以目前来看，针对晚期EGFR阳性的NSCLC患者，EGFR-TKIs联合放疗是一种颇有前景和未来的组合方式，值得我们进一步挖掘探索。

我们通过对真实世界数据的回顾性分析发现：晚期EGFR阳性NSCLC患者PD-L1表达水平存在差异，初诊时血液CEA水平、合并脑转移、肺癌细胞病理形态学分类对PD-L1表达影响较大，实体为主型患者更容易出现PD-L1高表达。EGFR-TKIs的近期疗效与EGFR突变亚型、联合治疗方式及PD-L1表达水平相关。PD-L1阳性表达的EGFR突变患者中，高表达的这部分患者更加值得我们注意。这部分患者EGFR-TKIs治疗疗效差获益少，所以在EGFR-TKIs治疗期间可能需要我们更加谨慎，尽可能缩短复查间隔时间，严密监测肿瘤病情，及时发现EGFR-TKIs耐药进展后及时更换治疗策略可能是更好的选择。这些发现提醒我们在临床实践中对这部分患者需要给予重点关注和个体化诊疗，也亟需大量前瞻性临床研究来提供更加充分严谨的循证医学证据。
